# Compartmentalized Structure of the Moderator Band Provides a Unique Substrate for Macroreentrant Ventricular Tachycardia

**DOI:** 10.1161/CIRCEP.117.005913

**Published:** 2018-07-31

**Authors:** Richard D. Walton, Ali Pashaei, Marine E. Martinez, Marion Constantin, Josselin Duchateau, Laura Bear, Caroline Cros, Caroline Pascarel-Auclerc, Yunbo Guo, David Benoist, Virginie Dubes, Ndeye Rokhaya Faye, Sebastien Chaigne, Sebastien Dupuis, Dominique Détaille, Line Pourtau, Philippe Pasdois, Fabien Brette, Julien Rogier, Louis Labrousse, Mélèze Hocini, Edward J. Vigmond, Michel Haïssaguerre, Olivier Bernus

**Affiliations:** 1 Université de Bordeaux, Centre de recherche Cardio-Thoracique de Bordeaux, U1045, France (R.D.W., A.P., M.E.M, M.C., J.D., L.B., C.C., C.P-A., Y.G., D.B., V.D., N.R.F., S.C., S.D., D.D., L.P., P.P., F.B., J.R., L.L., M.H., M.H., O.B.); 2 INSERM, Centre de recherche Cardio-Thoracique de Bordeaux, U1045, France (R.D.W.,A.P., M.E.M, M.C., J.D., L.B., C.C., C.P-A., Y.G., D.B., V.D., N.R.F., S.C., S.D., D.D., L.P., P.P., F.B., J.R., L.L., M.H., M.H., O.B.); 3 IHU Liryc, Electrophysiology and Heart Modeling Institute, Fondation Bordeaux Université, Pessac-Bordeaux, France (R.D.W., A.P., M.E.M, M.C., J.D., L.B., C.C., C.P-A., Y.G., D.B., V.D., N.R.F., S.C., S.D., D.D., L.P., P.P., F.B., J.R., L.L., M.H., E.J.V., M.H., O.B.); 4 Bordeaux University Hospital (CHU), Electrophysiology and Ablation Unit, F-33600 Pessac, France (J.D., J.R., L.L., M.H., M.H.); 5 Université de Bordeaux, Bordeaux Mathematics Institute UMR5251, France (E.J.V.); 6 Department of Electrical and Computer Engineering, University of Calgary, AB, Canada (E.J.V.).

**Keywords:** action potentials, heart ventricles, papillary muscles, Purkinje fibers, tachycardia, ventricular

## Abstract

Supplemental Digital Content is available in the text.

**Background:**

Papillary muscles are an important source of ventricular tachycardia (VT). Yet little is known about the role of the right ventricular (RV) endocavity structure, the moderator band (MB). The aim of this study was to determine the characteristics of the MB that may predispose to arrhythmia substrates.

**Methods:**

Ventricular wedge preparations with intact MBs were studied from humans (n=2) and sheep (n=15; 40–50 kg). RV endocardium was optically mapped, and electrical recordings were measured along the MB and septum. S1S2 pacing of the RV free wall, MB, or combined S1-RV S2-MB sites were assessed. Human (n=2) and sheep (n=4) MB tissue constituents were assessed histologically.

**Results:**

The MB structure was remarkably organized as 2 excitable, yet uncoupled compartments of myocardium and Purkinje. In humans, action potential duration heterogeneity between MB and RV myocardium was found (324.6±12.0 versus 364.0±8.4 ms; *P*<0.0001). S1S2-MB pacing induced unidirectional propagation via MB myocardium, permitting sustained macroreentrant VT. In sheep, the incidence of VT for RV, MB, and S1-RV S2-MB pacing was 1.3%, 5.1%, and 10.3%. Severing the MB led to VT termination, confirming a primary arrhythmic role. Inducible preparations had shorter action potential duration in the MB than RV (259.3±45.2 versus 300.7±38.5 ms; *P*<0.05), whereas noninducible preparations showed no difference (312.0±30.3 versus 310.0±24.6 ms, respectively).

**Conclusions:**

The MB presents anatomic and electrical compartmentalization between myocardium and Purkinje fibers, providing a substrate for macroreentry. The vulnerability to sustain VT via this mechanism is dependent on MB structure and action potential duration gradients between the RV free wall and MB.

**WHAT IS KNOWN?**

- The right ventricle is the dominant origin of ventricular arrhythmias in idiopathic cases.
- The moderator band, harboring part of the right Purkinje system, is a known origin of premature ventricular contractions, but its structural complexity and electrical behavior are poorly understood.

**WHAT THE STUDY ADDS?**

- The moderator band forms a substrate and potential therapeutic target through ablation for idiopathic ventricular tachyarrhythmia in the right ventricle.
- The myocardial compartment of the moderator band can sustain macroreentrant tachycardia through a circuitous pathway formed by bridging the right ventricular free wall and septum.
- Large diameter, absence of branching, and short activation-recovery intervals relative to the right ventricular free wall increase the potential role as substrate of the moderator band to macroreentrant ventricular tachycardia.

Ventricular tachyarrhythmias are a major cause of sudden cardiac death,^1–3^ notably in patients with structural heart disease. In normal hearts, ventricular tachycardia (VT) and premature ventricular contractions are typically originating from the right ventricular (RV) outflow tract^4,5^ or endocavity structures, such as free-running Purkinje fibers and papillary muscles.^6^ The moderator band (MB) plays an important structural role in the RV in humans through association with the papillary muscle. It is a known origin of premature ventricular contractions-induced tachyarrhythmias, which are characterized by a left bundle branch block–like morphology.^7^ But, its role as a substrate for maintenance of VT has not yet been evaluated.

The MB is a free-running muscular structure extending from the anterior septum to the anterior papillary muscle of the RV free wall of large mammalian species,^8,9^ including humans.^6^ The MB is highly organized and numerous in cardiac myocytes^9^ and consequently was initially thought to moderate RV distension. However, the MB was later found to support a major branch of the RV conduction network^8^ enabling rapid activation of the midfree wall of the RV.^10^ Preparations of isolated MB from pig have shown that the muscular compartment is sufficiently well coupled and organized to permit electrical propagation.^11^ However, intracompartmental coupling as well as electrical propagation kinetics, repolarization heterogeneities, and rate-dependence between the MB and the free wall and septum have yet to be investigated.

Here, the role of the MB in a novel macroreentrant VT circuit in human hearts is shown, and a detailed mechanistic investigation of the extent of dynamic electrophysiological interactions between the MB and ventricular myocardium is characterized in a large animal sheep model. Numeric simulations in intact hearts are used to support experimental findings.

## Methods

The data and analytic methods and study materials will be made available to other researchers for purposes of reproducing the results or replicating the procedure.

### Tissue Acquisition

Procurement and use of human donor hearts with informed consent from family members were approved by the National Biomedical Agency and in a manner conforming to the declaration of Helsinki. Donor hearts were procured at the Bordeaux University Hospital and transported in ice cold cardioplegia to the laboratory. Donor information are shown in Table I in the Data Supplement.

Hearts were obtained from sheep (n=16) weighing 40 to 50 kg in accordance with the guidelines from Directive 2010/63/EU of the European Parliament on the protection of animals used for scientific purposes and the local ethical committee. Sheep were premedicated with ketamine (20 mg/kg) and acepromazine (0.02 mL/kg), and anesthesia was induced by propofol (2 mg/kg) and maintained under isoflurane, 2%, in air/O_2_ (50/50%) after intratracheal intubation. Sheep were euthanized by intravenous injection with pentobarbital (30 mL/50 kg), and hearts were rapidly excised, cannulated, and flushed with cardioplegic solution, containing (mmol/L) NaCl, 110; CaCl_2_, 1.2; KCl, 16; MgCl_2_, 16; NaHCO_3_, 10; and glucose, 9.01 at 4°C.

### Histology of Human and Sheep MBs

Tissue samples (n=4 for sheep, n=1 for human) were stained for muscle fibers, nuclei, and collagen using standard Masson trichrome staining. See materials in the Data Supplement for a detailed description.

### Preparations of Human and Sheep Myocardium

Dual coronary-perfused ventricular wedges (n=12 for sheep, n=2 for humans) were prepared by dissecting the anterior left ventricle and RV extending from the lateral left ventricular free wall to the posterior RV border while keeping the anterior two-thirds of the interventricular septum (Figure I in the Data Supplement). Both the left and right ostia of the coronary circulation from the aortic root were cannulated and perfused. Perfusion leaks at cut surfaces were carefully tied-off and preparations mounted on to a frame, exposing the endocardial surface of the RV. Wedges were submersed and perfused with a saline solution containing (mmol/L) NaCl, 130; NaHCO_3_, 24; NH_2_PO_4_, 1.2; MgCl_2_, 1; glucose, 5.6; KCl, 4; CaCl_2_, 1.8, at 37°C and pH7.4.

### Optical and Electrical Mapping

Preparations were imaged using optical mapping of the RV endocardial surface after being mechanically uncoupled using 15 µmol/L blebbistatin and loaded with the voltage-sensitive dye, Di-4-ANBDQBS. The endocardial surface was illuminated with monochromatic light emitting diodes at 627 nm (Cairn Research Ltd, Kent, United Kingdom). Optical images (100×100 pixels) of signals passed through a 715-nm long-pass filter were acquired using a Micam Ultima CMOS camera (SciMedia USA Ltd, CA) at 2 kHz with a spatial resolution of 0.7×0.7 mm. Optical signals were filtered using a low-pass frequency filter at 120 Hz followed by spatial averaging (kernel 2.1 mm) and temporal averaging (kernel 1.5 ms).

Pseudo-ECG recordings across tissue preparations were recorded throughout experiments. In addition, to record activity along the MB and septum, unipolar electrograms were recorded from 5 locations simultaneously: (1) septum, close to the MB attachment; (2) proximal (septal) end of the MB; (3) mid-MB; (4) distal (RV) end of the MB; and (5) the anterio-lateral papillary muscle. A reference electrode was positioned in the bath far from recording electrodes. All electrical recordings were acquired at 10 kHz, and signals were treated by a forward-backward Butterworth filter with a 120 Hz low-pass cutoff.

### Pacing Protocols

Two bipolar stimulation locations were used: endocardial surface of the RV midwall and the MB at approximately half of its length. Measurements of activation latency at varying stimulation currents were recorded at a basic cycle length of 2 Hz. S1S2 pacing was assessed for each pacing site and a combined S1RV-S2MB protocol at twice the stimulation threshold. The stimulation sequence consisted of a train of 15 S1 stimuli at a basic cycle length of 2 Hz followed by S2. Coupling intervals were adjusted incrementally from the effective refractory period (ERP) 6× by 5 ms, 2× by 10 ms, 2× by 25 ms, and 2 increments of 50 ms. In humans, VT was induced by burst pacing at 30 Hz from MB electrodes. In addition, to mimick cardiomyopathic disease, the VT-inducible human case was further perfused with flecainide, 15 µmol/L, and burst pacing was retested.

### Analyses and Statistics

VT involving the MB was defined as ≥3 cycles with unidirectional propagation along the MB and activation of the RV free wall originating from the insertion of the MB. Dominant frequencies and regulatory indices of optical signals were computed (from a frequency window of 0–12 Hz) over the duration of the VT acquired. Statistical differences were assessed by the Student *t* test, Mann-Whitney test, or Wilcoxon matched-pairs signed-rank test and defined by *P<0.05 and ****P<0.0001.

### In Silico Reentry Study

A detailed description of computer modeling in this study is found in the materials in the Data Supplement. Briefly, we used a realistic 3-dimensional rabbit ventricle finite element mesh with anisotropic fibers and Purkinje system (see Figure II in the Data Supplement) that we have previously developed.^12–14^ An MB was manually added to the geometry. Table II in the Data Supplement details the changes and lists modifications for each ionic model parameter that were applied to adjust for modeling the effective heart size of sheep within a rabbit ventricular geometry (see Table III in the Data Supplement) for better correspondence with experiments.^14^ Pacing protocols were applied to mimic experiments.

## Results

### Structural Substrate of the MB

**Figure 1.**
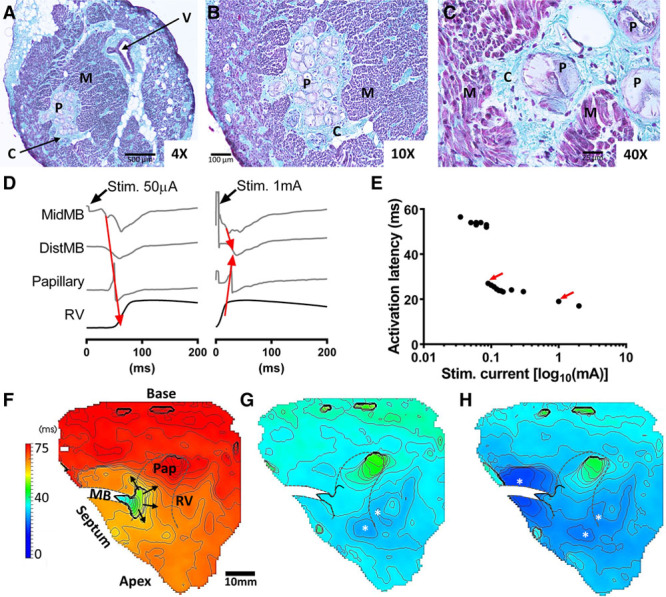
**Structural and functional compartmentalization of the moderator band (MB) from sheep.** Transverse cross-sections of the MB at 4× (**A**), 10× (**B**), and 40× (C). Cellular and extracellular constituents are collagen, C; lipid droplets, L; myocardium, M; Purkinje fibers, P; and vasculature, V. **D**, MB pacing at 50 and 1000 µA stimuli currents. Red arrows indicate the direction of propagation. **E**, Step-wise changes (red arrows) of activation latency of the right ventricular (RV) free wall with stimulation current. Activation time maps of the RV free wall are shown for 35 µA (**F**), 90 µA (**G**), and 1 mA (**H**). Focal activation sites are indicated by *. Stimulus occurred at t=0 s.

The MB structure is composed of 2 excitable, yet uncoupled compartments in sheep. Purkinje fibers were separated from the myocardium in a coaxial configuration by lipid deposits and extensive extracellular collagen surrounding individual bundles, as seen in transverse cross-sections of the MB (Figure 1A–1C) and insertions (Figure III in the Data Supplement) with no apparent coupling. The Purkinje fiber/muscle ratios were highly variable, ranging from 0.04 to 0.63 (Figure IV in the Data Supplement). This may be explained by a high intraspecies variability of the MB thicknesses (3.3±0.9 mm, minimum=2.1 mm, maximum=5.0 mm, in sheep).

### Activation Patterns After MB Pacing

Figure 1D shows electrical recordings of the myocardial compartment of the MB and optical recordings at the MB insertion with the RV free wall. Stimulation at the current threshold (35 µA) for one-to-one capture of the MB induced continuous propagation toward the RV free wall. The recording of the mid-MB shows early local activation of the midportion of the MB, followed by far-field activation of the RV free wall. On substantial increases in stimulus current (ie, 1 mA), the site of earliest activation shifted to the RV free wall, consistent with capturing the fast propagating Purkinje fibers. Incremental changes in stimulus current developed nonlinear shortening of activation latency of the RV free wall whereby increased stimulation current caused step-wise reduction of activation latency (Figure 1E). Low stimulus current of 50 µA resulted in relatively slow wave propagation emanating from the base of the MB to the RV free wall (Figure 1F). At 90 µA, corresponding to the first step-wise reduction of RV activation latency, 2 sites of focal activation were observed in the RV free wall distant to the base of the MB (Figure 1G). A third remote focal site of activation in the RV occurred at 1 mA (Figure 1H). Long activation latencies in the RV were attributed to conduction along the muscular compartment of the MB, and short activation latencies were attributed to capturing Purkinje.

Conduction delays between the RV free wall and MB myocardium in sheep were dependent on the direction of propagation across the MB insertion and the coupling interval (Figure V in the Data Supplement). This was determined through S1S2 pacing of either the RV free wall or the myocardial compartment of the MB. Across all experiments, activation latency of the MB after RV stimulation at the S1 basic cycle length was 12.7±6.1 ms. This was increased to 16.0±8.3 ms at the ERP of the stimulation site. For MB stimulation, activation latency of the RV was 14.4±13.6 ms at the basic cycle length but significantly prolonged to 32.5±16.7 ms at the ERP (*P*<0.05).

### Macroreentry Involving the Human MB

**Figure 2.**
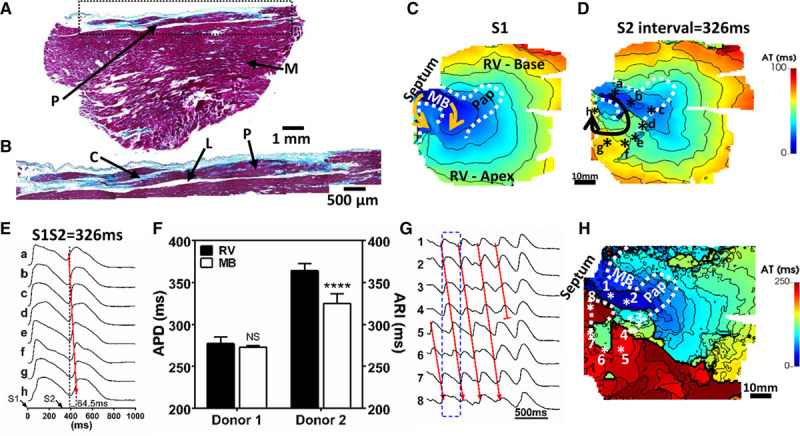
**Macroreentrant ventricular tachycardia (VT) involving the human moderator band (MB).** Histological sections of the MB from donor no. 4. Montages of MB cross-sections along its longitudinal axis at 4× (**A**) and 10× (**B**). Labels are the same as for Figure 1. Activation patterns during pacing human donor no. 2 MB at (**C**) basic cycle lengths of 500 ms and (**D**) a short coupled beat of 326 ms. **E**, Optical action potential traces of the final S1 and S2 pulses are extracted along a circuitous pathway involving the MB (sites a–c shown in **D**), MB insertion to the free wall (d, e), right ventricular (RV) free wall (f, g), and septum (h). Short coupled beats showed unidirectional activation and enhanced activation time (AT) gradients between points a and h, relative to S1 pacing. **F**, Comparison of mean action potential duration (APD) and activation-recovery interval (ARI) for RV and MB, respectively, between 2 human donor hearts. **G**, Nonsustained macroreentrant VT from the circuitous pathway shown in **D**. **H**, Activation pattern during macroreentrant VT derived from the time window shown in (**E**; dashed blue box). Statistical comparisons were performed using a Mann-Whitney test, ****P<0.0001 and NS, not significant.

As observed in sheep, the human MB also contains Purkinje fiber branches surrounded by thick endomysial collagen sheaths, further separated from the muscular compartment by adipose tissue (Figure 2A and 2B). However, a greater muscular content was observed in the human MB. Conduction behavior and repolarization heterogeneity were examined from 2 human wedge preparations with intact MBs. MB thicknesses from human preparations were 4.0 and 8.5 mm in donor no. 1 and donor no. 2, respectively. Pacing at a basic cycle length of 500 ms on the MB triggered activation propagating bidirectionally toward both septal and RV free wall insertions of the MB (Figure 2C). The activation latency of the RV when pacing MB was 10.8 and 17.9 ms, respectively (see Figure VI in the Data Supplement). In donor no. 2, a sufficiently short coupled stimulation at the same site resulted in unidirectional propagation toward the RV free wall to excite the septum from the RV free wall instead of the MB directly (Figure 2D). A circuitous pathway of activation along the MB, anteriorly across the RV free wall, and in to the septum was observed through optical action potentials (APs) (Figure 2E). Relative to the MB, the activation time of the septum was 64.5 versus 9.4 ms with bidirectional propagation. Furthermore, a pronounced gradient of action potential duration (APD) between the MB and RV, with longest APD in the RV, was found in donor no. 2 but absent in donor no. 1 (Figure 2F).

Four repeat trials of burst pacing of the MB led to MB-mediated macroreentrant VT with mean cycle lengths of 277.9±15.1 ms. Figure 2G shows example optical mapping traces along the reentrant circuit during the arrhythmia, consistent with those observed during short coupled activation from the same preparation. Figure 2H shows the activation sequence of the VT originating and exiting from the MB. Conduction slowing at the septo-apical region provided the delay of activation for re-excitation of the MB from the septum. VT termination coincided with wavefront collision with the repolarization wave (wave back) along this macroreentrant circuit at the site of the RV-MB insertion, thus further supporting this circuit trajectory in the maintenance of VT. Macroreentrant VT could not be induced in donor no. 1, which may be related to a lack of APD heterogeneity between the MB and RV (Figure 2F).

**Figure 3.**
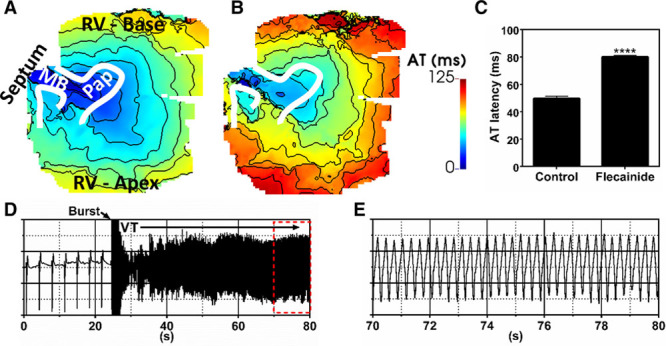
**Sustained macroreentrant ventricular tachycardia (VT) sustained by flecainide in human.** Activation patterns in the absence (**A**) and presence (**B**) of flecainide, 5 µmol/L after stimulation of the moderator band (MB) in human donor no. 2. Activation latency of the right ventricular (RV) free wall, measured from the same 5×5 pixel region in the RV free wall, was prolonged by flecainide. **C**, Sustained macroreentrant VT after burst pacing in the presence of flecainide was established. **D**, A pseudo-ECG during the induction and maintenance of macroreentrant VT with flecainide. **E**, Enlargement of the red dashed box in (**F**). Statistical differences were determined by the Mann-Whitney test. ****P<0.0001. AT indicates activation time.

Supplementing the tissue perfusate with flecainide, 5 µmol/L, reduced conduction velocity and increased total activation time of the preparation (Figure 3A and 3B). Activation latency of the RV free wall when pacing the MB was significantly prolonged (Figure 3C). During flecainide treatment, sustained VT could be induced through burst pacing of the MB (Figure 3D). The anatomic stability of VT was clearly shown by the monomorphic pattern identified on the ECG (Figure 3E). Mean cycle length of VT across all pixels determined from a window of 15 seconds was 299.2±24.4 ms, significantly longer than that observed just before flecainide infusion (270.0±28.1 ms; *P*<0.0001).

### Sheep MB Supports VT

**Figure 4.**
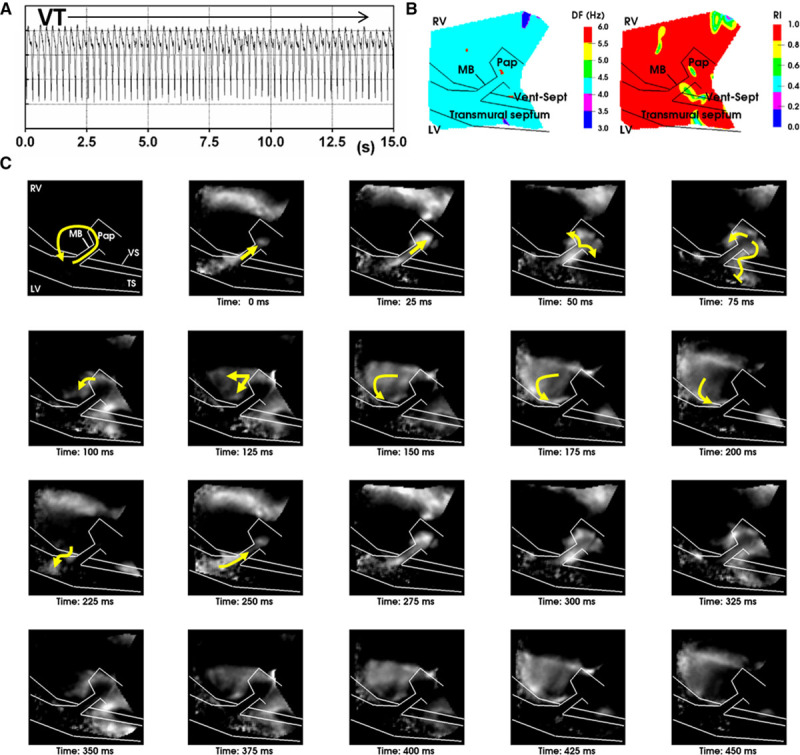
**Sustained macroreentry involving the moderator band (MB) in sheep.** **A**, PsuedoECG of sustained ventricular tachycardia (VT) triggered by a short coupled S2 with interval of 282 ms in sheep. **B**, Dominant frequency and regularity indices of optical recordings of macroreentrant VT. **C**, Frame-shots of a movie of optical action potential derivatives show wavefront propagation throughout 2 complete and consecutive cycles of macroreentry. Yellow arrows indicate wavefront orientation for 1 complete macroreentry circuit. RV indicates right ventricular; TS, transmural cut surface; and VS, ventricular-septal groove.

**Figure 5.**
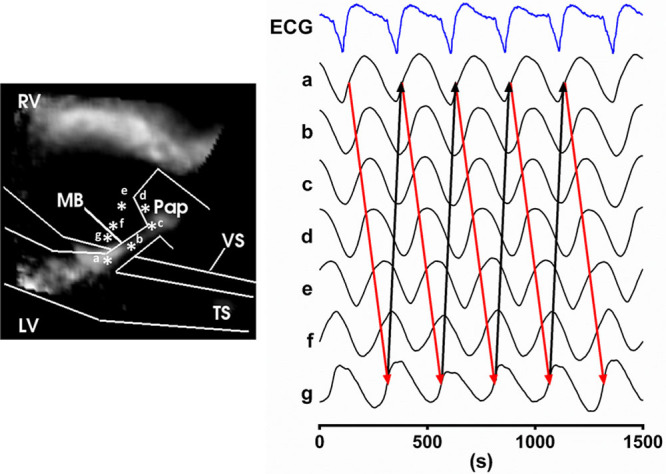
**The macroreentry circuit.** Optical action potential traces derived from positions indicated by * in the wavefront snap-shot image. Red arrows show the direction of propagation, and black arrows indicate re-excitation of the moderator band (MB) and initiation of the following macroreentrant ventricular tachycardia (VT) cycle. LV indicates left ventricular; RV, right ventricular; TS, transmural cut surface; and VS, ventricular-septal groove.

**Figure 6.**
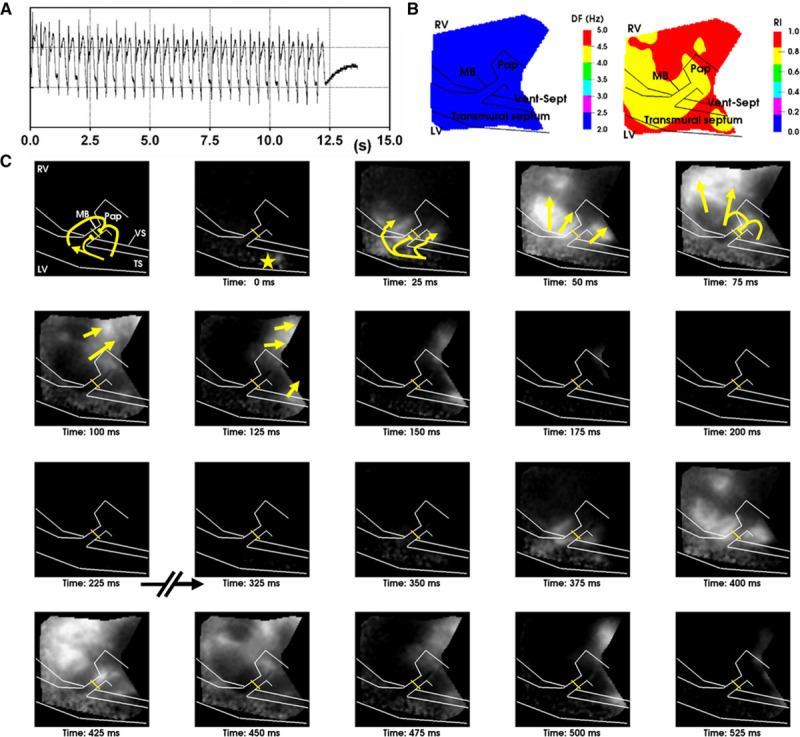
**Cutting the moderator band (MB) in sheep.** **A**, PsuedoECG during and after self-termination of ectopic activity with sources independent of the MB. **B**, Dominant frequency and regularity indices of optical recordings of focal activity. **C**, Frame-shots of a movie of optical AP derivatives show wavefront propagation throughout 2 complete and consecutive spontaneous focal discharges. Yellow arrows indicate wavefront orientation for 1 complete spontaneous beat. LV indicates left ventricular; and RV, right ventricular.

Next, we verified the occurrence of VT in the sheep ventricles, which allowed us to investigate its mechanisms in more detail. In total, 14 incidences of VT were observed across the different hearts: 8 were sustained and 6 terminated after 7.3±8.5 seconds. The example in Figure 4 shows sustained monomorphic VT with a mean dominant frequency across the image plane of 4.0±0.2 Hz (mean cycle length=244.4±17.3 ms). The VT episode was stable and organized, indicated by a spatial mean regularity index of 0.92±0.1 (Figure 4B). VT was macroreentrant, characterized by unidirectional propagation along the MB from septum to RV (as seen by the activation wavefront sequence shown in Figure 4C). The wavefront exited the MB in both apical and basal directions of the RV free wall. The apical portion of the wavefront propagated more rapidly in to the septum, but conduction was blocked near to the entry site of the MB where it collided with its wave tail. However, conduction through the basal part of the septum was sufficiently delayed for recovery and re-excitation of the MB during diastole. Meanwhile, the remainder of the wavefront passively propagates toward the posterior-apical RV, activating the bulk of the myocardium, as observed by comparing the pseudo-ECG with local optical AP signals (Figure 5). This macroreentrant circuit was highly consistent and conserved throughout the episode. In this example, the MB was severed after VT induction, causing termination of the macroreentry. On reinduction, a secondary arrhythmic substrate was observed, but it was ectopic, with distinct diastolic pauses, and nonsustained, terminating after 12.3 seconds (Figure 6A). Furthermore, Figure 6B shows the dominant frequencies (2.5±0.0 Hz), and regularity indices (0.84±0.04) of the secondary substrate were less than for the primary MB-mediated VT (*P*<0.0001). Figure 6C shows a time series for the secondary arrhythmic substrate after severing of the MB. A septal focal source, distant from the MB, activates the septum and proximal MB and then in turn propagates in to the RV free wall and distal MB. Propagation along the MB occurred from the septal and RV insertions independently as a result of cutting the MB at mid length.

### Underlying Electrophysiological Substrates of VT

**Figure 7.**
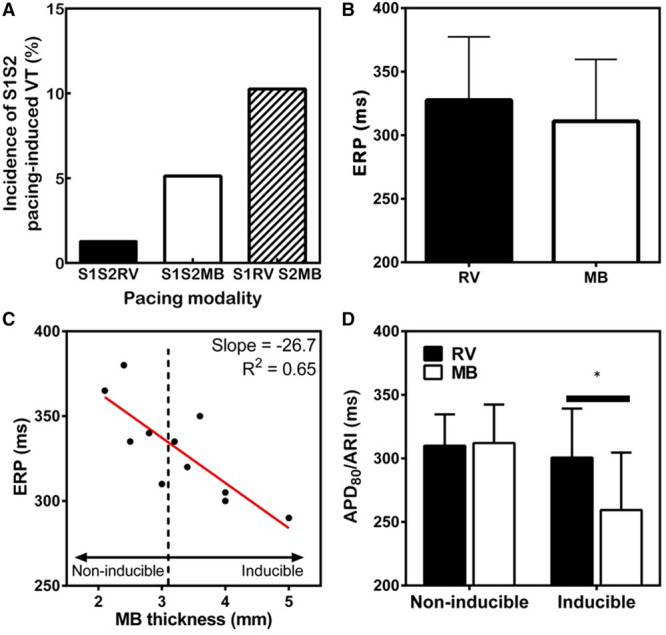
**Ventricular tachycardia (VT) inducibility is linked to moderator band (MB)-right ventricular (RV) gradients of refractoriness.** **A**, The frequency of VT induced by S1S2 pacing configurations. **B**, Comparisons of effective refractory period (ERP) between the MB and RV free wall. **C**, Linear correlation of ERP at the MB and MB thickness. **D**, Data were separated in to inducible and noninducible for VT during S1S2 pacing. Action potential duration (APD) for optical recordings and activation-recovery interval (ARI) for unipolar recordings were compared for 2 groups at the basic S1 pacing cycle length. Statistical differences were determined by the Wilcoxon matched-pairs signed-rank test. *P<0.05, n=12.

To further understand the electrophysiological substrate of the MB during VT, ERP and repolarization properties of the MB were assessed. VT was observed across all pacing protocols in sheep; however, the incidence of VT was 2-fold greater for S1S2MB and 8-fold greater for S1RV-S2MB (Figure 7A). Measurements across all experiments showed that ERP was not significantly different between the MB and RV (Figure 7B). However, considering the variability of MB thickness, complexity of the MB macrostructure (Figure 1), and variability of muscle/Purkinje fiber ratios (Figure IV in the Data Supplement), the ERP may be influenced by variations of electrotonic loading, which is sensitive to tissue mass^15^ and coupling.^16^ Therefore, ERP of the MB was compared against MB thickness (Figure 7C). A strong linear relationship was identified, whereby thicker MBs were associated with shorter ERP. It was found that the vulnerability for VT to S1S2 stimulation was also dependent on MB thickness. Inducible preparations had MB thicknesses ≥3.2 mm. A significant difference between ERP of the RV and MB was revealed for inducible (303.3±45.0 versus 334.4±25.3 ms; *P*=0.015) but absent in noninducible experiments (322.5±17.6 versus 348.8±59.7 ms; *P*>0.05). The difference in ERP was associated with a concordant short activation-recovery interval in the MB of inducible preparations (86%±15%) versus RV (Figure 7D). No difference was observed in noninducible preparations.

### Simulations of VT

**Figure 8.**
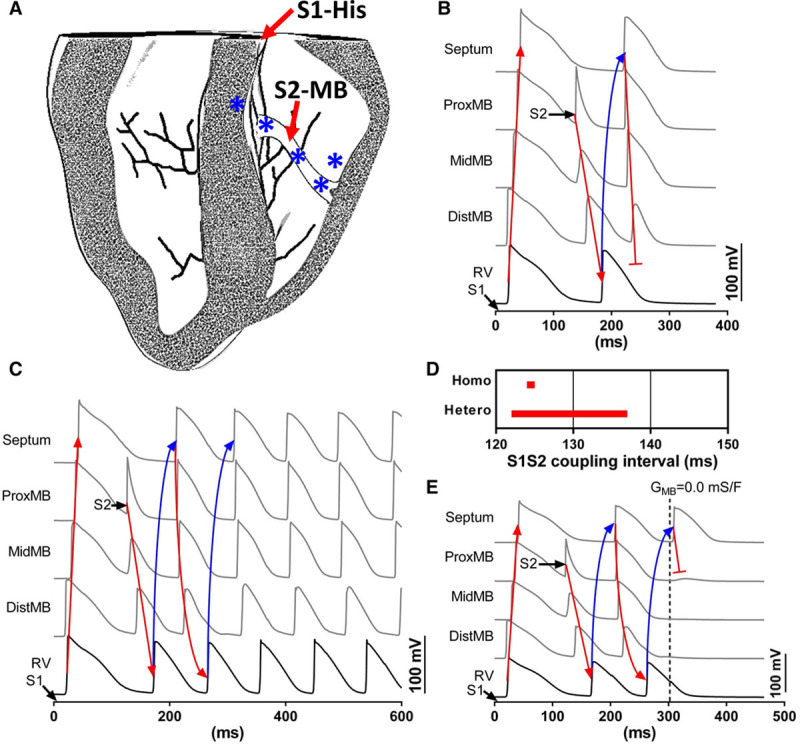
**Simulations of the vulnerability to ventricular tachycardia (VT).** Sinus rhythm in a whole ventricle model was simulated with a short coupled S2 pulses from the moderator band (MB). **A**, Cross-section of the whole heart geometry. Simulations of S1S2 pacing in models with either homogeneous ionic properties in the myocardium (**B**) or heterogeneous short action potential duration (APD) in the myocardial compartment of the MB (**C**). Nodes where electrical AP traces were extracted for comparison are indicated by * in **A** and correspond with locations in experiments (Figures 4 and 5D). Directions of propagation and reentry are indicated with red and blue arrows, respectively. **D**, Windows of vulnerability of VT throughout S1S2 coupling intervals. **E**, The conduction of the MB was blocked during reentry by the reduction of *G*_*Na*_ t zero in MB myocardial nodes at t=300 ms. Locations of traces are as for **B** and **C**.

Further understanding of the mechanism underlying VT observed in experiments was assessed in computer models using similar pacing protocols. Furthermore, models permitted investigation of the effects of premature beats arising from the MB during sinus rhythm, which was not feasible in experimental wedge preparations. VT susceptibility was assessed initially in a homogeneous model with respect to differences of ionic properties between the MB and myocardium. The Purkinje network was paced at the proximal end of the His bundle during S1 stimulation (Figure 8A; Movie I in the Data Supplement). S2 stimulation of the muscular compartment of the MB propagated preferentially in the anterograde direction in to the RV free wall. Reactivation of the MB could be achieved from the septal insertion of the MB; however, functional block at the insertion with the RV free wall terminated the wavefront because of insufficient loading capacity of the distal MB, shown by a reduced AP amplitude (Figure 8B).

The same protocol was assessed in the heterogeneous model, whereby APD of MB muscle was reduced by 21% to mimic experimental observations. Figure 8C and Movie II in the Data Supplement show that reentry in to the MB after S2 persisted by exiting in to the RV free wall and repetitive reactivation of the MB from the septum gave rise to a sustained macroreentrant circuit. Figure 8D shows the window of vulnerability of S1S2 pacing intervals to induce VT was considerably greater in the heterogeneous model (16 versus 2 ms). The role of the MB as substrate for macroreentrant arrhythmia was demonstrated by blocking conduction through the MB after the arrhythmia was established and at a moment when the MB was in a repolarized state (t=300 ms; Figure 8E; Movie III in the Data Supplement). The existing wavefront could no longer re-excite the MB, preventing further reentry. Simulations also permitted further assessment not feasible in experiments, including the impact of using wedge preparations versus whole hearts and the presence of the Purkinje network. Simulations were performed using S1 pulses of the RV free wall and MB (see materials in the Data Supplement and Movies IV–VI in the Data Supplement), in the absence of a Purkinje network (Movie VII in the Data Supplement), and in a wedge preparation equivalent to experiments (Movie VIII in the Data Supplement). Whether the model used a whole heart of wedge preparation geometry, macroreentrant VT was inducible and maintained. Similarly, removing the Purkinje network did not modulate the ability to induce macroreentrant VT. Furthermore, the absence of the Purkinje network resulted in increased stability of the reentry, consolidating the role of the myocardial compartment of the MB in the macroreentrant circuit in simulations.

## Discussion

### Novel VT Macroreentrant Mechanism

Macroreentry is a well-established mechanism for VT.^17,18^ It is characterized by a recurrent circus activation around an inexcitable obstacle or anatomic feature. For this to occur, a tract (or isthmus) of myocardium that is insulated from the surrounding conductible tissue, except for an entry and exit site, is required to permit re-excitation of the bulk myocardium. Conduction along the isthmus, therefore, must be sufficiently slow or long as to allow recovery of the myocardium at the exit site. Such conditions explain that macroreentry in the ventricles is largely most commonly observed in pathological myocardium, particularly channels of surviving myocardium in an infarct or cardiomyopathic zone.^17^ To our knowledge, in normal hearts in humans, only the left Purkinje fascicles have been demonstrated to participate in macroreentrant VT,^17–20^ but the complete circuit is still not fully understood.

The present study identified a novel macroreentry circuit through the myocardial compartment of the MB in the structurally normal RV of humans that was recapitulated in sheep. With proximal and distal insertions at the anterior septal and RV papillary muscles, the MB provides a conducting link between these intracavity structures and a circuitous pathway anteriorly along the RV free wall and posteriorly within septum. Macroreentry was established on premature activation of the MB and unidirectional propagation toward, and eliciting activation of, the RV free wall. Re-excitation of the MB at the septal end provided the isthmus for reentry in to the RV free wall (see Figure 8B for a schematic representation). Macroreentry was inducible and maintained preferentially in a subset of sheep with heterogeneous APDs between the MB and RV free wall. Shortening of the APD in combination with conduction velocity restitution reduced the wavelength for excitation, providing the substrate for macroreentry. In support, simulations also showed that a shorter APD in the MB increased the vulnerability window for VT.

### Conduction in the MB

The unique coaxial structure of the MB contains a rapid conduction pathway via Purkinje fibers surrounded by a relatively slower myocardial pathway. We demonstrated that the myocardial compartment of the MB could be activated independently of the Purkinje (Figure 1). Histological assessment of both sheep and human MB identified common structural features, including a thick collagenous sheath surrounding Purkinje fiber bundles and an absence of abutting myocardium or transitional cells, which form the junctioning termini of Purkinje to the myocardium.^21^ Therefore, relatively slow propagation of wave fronts within the myocardial compartment could be tolerated without preexcitation of the downstream tissue in the RV free wall of sheep. In conjunction, the relatively long free-running path length of the MB (2.3±0.25 cm) provided a means for substantial latency of activation of the RV free wall, fulfilling prerequisites for the maintenance of reentry and its role as substrate. The length of the MB in humans was more variable than sheep with mean±SD of 1.7±0.70 cm. But, the inducible human heart was that with the longest MB (2.4 cm).

High stimulation currents, however, could capture embedded Purkinje fibers within the MB (Figure 3). Fast conduction of the Purkinje fibers led to rapid preexcitation of the RV free wall at Purkinje-muscle junctions and led to retrograde activation of the distal MB (Figure 8A), akin to reflected propagation emanating from the Purkinje-muscle junction.^22^

### Dynamic Interplay of Conduction and Repolarization Gradients

VT was established predominantly from short delays of premature activation of the MB, near to the ERP. The direction of propagation during S1 determined the gradient of repolarization time (Figure 6). A sufficiently short coupled S2 stimulus could subsequently trigger unidirectional propagation toward early repolarized tissue. Inducibility of VT was further determined by repolarization heterogeneity between the MB and RV (Figure 6). The safety factor for conduction decreases when propagating from thin to larger well coupled structures.^23^ Therefore, the shorter ERP (longer diastolic interval) of the heterogeneous models likely enabled sufficient reactivation of Na^+^ current in distal MB cells to overcome high electrotonic loading at the insertion, thus achieving conduction from the MB to the RV free wall and complete reentry.

### MB Provides Both Structural and Functional Substrate for VT

This study also demonstrated that MB thickening was associated with short ERP and an increased propensity for arrhythmias in both human and sheep. MB thickness and myocardial content were variable (see Figure IV in the Data Supplement), but thickening showed increased myocardial density and increased cellular/extracellular ratios. Noncellular constituents, such as lipid deposits, were also identified and likely further modulates loading conditions for conduction.^24^

### Clinical Implications

Here, a novel role for the MB as substrate for macroreentrant VT was shown. Potentially, a similar mechanism including coaxial compartments may be present in other structures of the heart. Notably in left Purkinje fascicular VTs, there is a fascicular component of the circuit (which is the most common and easily identifiable target for ablation) while the verapamil-sensitive slow component is largely unknown.^20^

Premature ventricular contractions have been reported to originate from RV papillary muscles^25,26^ and the MB,^6,7^ as well as some VTs. Such cases are described as focal, but the resolution of current clinical catheters (commonly a 7-mm distal bipole) may be insufficient to differentiate intricate myocardial-Purkinje structures. The markers that increase the susceptibility to VT maintenance by the MB are increased MB thickness and shorter ERPs, which are suggestive of this mechanism identified in humans. A retrospective study of patients with VT by Abouezzeddine et al^6^ identified 7 of 190 patients (3.8%) presenting VT where radiofrequency ablation was targeted to the MB. Interestingly, the cohort of patients for MB origins of VT was larger than that of other endocavity structures, including papillary muscles (5 patients) and free-running Purkinje fibers (3 patients). Importantly, the mean MB thickness in humans reported previously and the thicknesses of those measured within this study were greater than the threshold of macroreentrant VT induction observed in sheep.

### Limitations

Experiments were performed in a novel ventricular wedge configuration to have a complete intact MB and simultaneous access to the endocardium. It is conceivable that reentry circuits may be impacted by removal of the posterior aspect of the ventricles. However, using simulations, we assessed the impact of the whole ventricle geometry compared with wedge geometry on macroreentrant VT via the MB. We observed little or no changes to either electrophysiological dynamics of the macroreentrant circuit or the vulnerability windows of VT. This study did not investigate trigger mechanisms of VT but provided mechanistic insight in to the role of the MB as substrate. The modality for the induction of macroreentry was either premature stimulation or burst pacing at the MB. Interestingly, premature stimulation did not induce sustained arrhythmias in human tissue. This is, however, consistent with modes of induction for reentrant arrhythmia in the clinic,^27^ where the use of catecholaminergic agents are often needed.^28^ Entrainment of the macroreentrant circuit was not performed as limited work space to avoid obstructing the imaging field, and stimulation sites were available around recording electrodes. However, selective pace mapping, a commonly applied alternative,^29^ was used to confirm the role of the MB in the mechanism for VT. In addition, the impact of conduction slowing by flecainide infusion and severing of the MB confirmed the role of the MB as primary substrate for macroreentry.

### Conclusions

The muscular compartment of the MB can provide a substrate for macroreentrant arrhythmias by abridging the RV free wall and septum in sheep and humans. The vulnerability to sustaining VT via this mechanism is dependent on MB structure, both length and thickness, and increasing APD gradients between the RV free wall and MB.

## Acknowledgments

Computer time for this study was provided by the computing facilities MCIA (Mésocentre de Calcul Intensif Aquitain) of the Université de Bordeaux and of the Université de Pau et des Pays de l’Adour. The authors thank Bruno Quesson, Bruno Stuyvers, Fanny Vaillant, Sabine Charron, Gilles Bru-Mercier, Emma Abell, Audrey Semont, and Pr Igor Efimov and team for their support in this study.

## Sources of Funding

This study received financial support from the French Government as part of the Investments of the Future program managed by the National Research Agency (ANR; grant number ANR-10-IAHU04-LIRYC). This work was also supported by the Marie Curie (grant numbers IEF-PSCD-300299 to Dr Walton, IRSES-CORDIS3D-317766 to Dr Bernus); and European Research Council (FP7/2007–2013 grant agreement number 322886 to Dr Haïssaguerre).

## Disclosures

None.

## Supplementary Material

SUPPLEMENTARY MATERIAL
