# Supplementary material for: Compartmentalized Structure of the Moderator Band Provides a Unique Substrate for Macroreentrant Ventricular Tachycardia
Source: Circ Arrhythm Electrophysiol. 2018 Jul 31;11(8):e005913. doi: 10.1161/CIRCEP.117.005913 (PMC7661046; doi:10.1161/CIRCEP.117.005913)
Supplement: SUPPLEMENTARY MATERIAL [file hae-11-e005913-s001.pdf]

SUPPLEMENTAL MATERIAL

Methods

Acquisition of human samples

Procurement and use of human donor hearts with informed consent from family members was approved by the National Biomedical Agency and in a manner conforming to the declaration of Helsinki. Donor hearts were procured at the Bordeaux University Hospital and transported in ice cold cardioplegia to the laboratory. Donor information are shown in Supplemental Table 1.

Supplemental table 1. Human donor information

Donor #	Sex	Age	Cause of death	Pharmacological treatments	Prior CV dysfunction	Comments
1	M	76	Stroke	Olmasartan, rabeprazole, domperidone, rasagiline, piroxicam, silodosin	None	Non-transplantation due to age
2	F	83	Stroke	Rivaroxaban, trimetazidine, nebivolol	Atrial fibrillation	Non-transplantation due to age

Abbreviations are: Male, M, Female, F and cardiovascular, CV.

Histology

Tissue samples were fixed in 4% paraformaldehyde dissolved in PBS, pH 7.4 and agitated during 48 hrs. Samples underwent serial dehydration steps of ethanol at 70%, 80%, 95% and 100%, followed by toluene, 100%, which permitted the penetration of paraffin (warmed to 56°C) in to the sample. Embedded samples were cooled to -4°C to accelerate solidification then stored at -20°C. Samples were sectioned at thicknesses of 8 µm using a microtome (Leica RM2255, France). Sections were treated with toluene to remove paraffin then rehydrated by serial dilutions of ethanol at 100%, 95%, 80%, followed by immersion in tap water. Sections were submitted to Masson's trichrome staining by immersing sections in: xylidine ponceau, 5 mins; rinsing with distilled water, 5 mins; Biebrich Scarlet-acid fuchsin solution, 1 min; 3% phosphomolybdic, 1 min; 1% glacial acetic acid, 1 min; 1% light green SF yellowish, 2 min and then 1% acetic acid, 1 min. Sections were again dehydrated by ethanol 95%, 6 mins and 100% ethanol, 6 mins followed by toluene, 3 mins and stored at room temperature in air. Sections were imaged using a standard binocular light microscope mounted with a high resolution CDD camera (Nikon DXM1200F, Japan).

Myocardial and Purkinje staining were quantified from histological sections using ImageJ. Purkinje and myocardium were masked and an RGB color threshold was applied to isolate myocardial-specific or Purkinje-specific staining from collagen. The quantity of each tissue component was quantified from the integral of frequency histograms of pixel intensity.

Experimental preparation of the sheep myocardium

Novel dual coronary-perfused ventricular wedges were prepared by dissecting the anterior left and right ventricles extending from the lateral left ventricular free wall to the posterior RV border while keeping the anterior two thirds of the interventricular septum (Supplemental figure 1). Both the left and right ostia of the coronary circulation from the aortic root were cannulated and perfused. Perfusion leaks at cut surfaces were carefully tied-off or cauterized under cold cardioplegic solution and preparations mounted on to a frame, exposing the endocardial surface of the RV. Wedges were submersed and perfused with a saline solution containing (mM): NaCl, 130; $NaHCO_3$, 24; NH_2PO_4, 1.2; $MgCl_2$, 1; glucose, 5.6; KCl, 4; $CaCl_2$, 1.8, at 37°C and pH7.4.

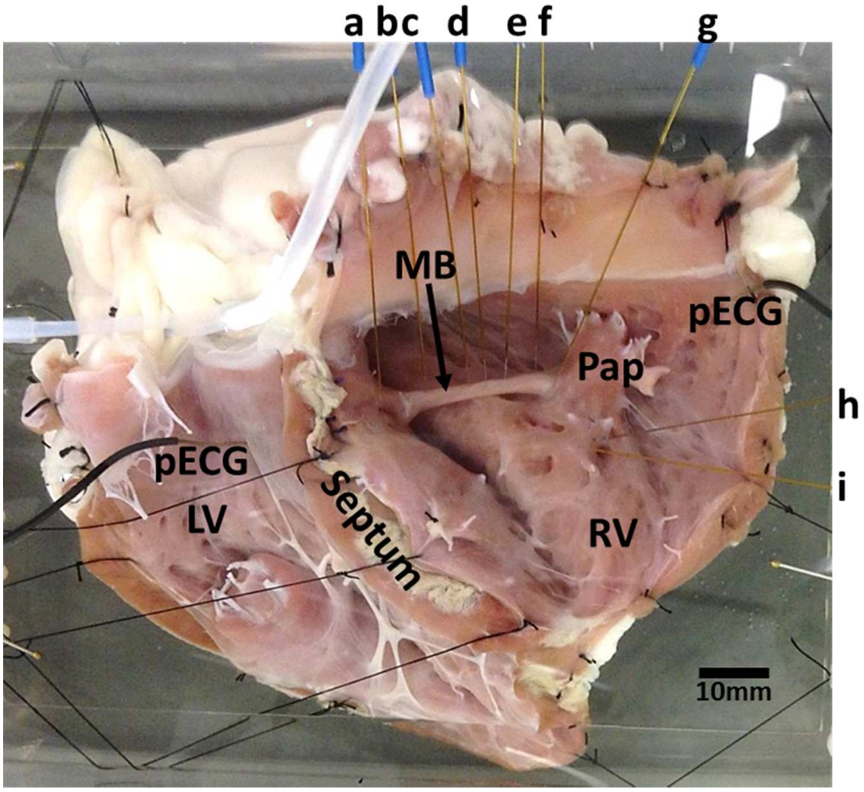

Supplemental figure 1. Novel intact MB preparation. Endocardial view of a dual-coronary perfused ventricular wedge preparation of the anterior heart. Electrode impalements are: a) Septum; b) proxMB; c,d) bipolar stimulation of MB; e) midMB; f) distMB; g) papillary; h,i) RV endocardial bipolar stimulation.

Optical and electrical mapping of the sheep myocardium

Novel dual coronary-perfused left anterior and right ventricular wedge preparations were imaged using optical mapping of the right ventricular endocardial surface. Preparations were loaded with the voltage-sensitive dye Di-4-ANBDQBS that was excited by illumination of the endocardial surface using monochromatic LEDs at 627 nm (Cairn Research Ltd, Kent, UK). Optical images (100x100 pixels) of signals passed through a 715nm long-pass filter were acquired using a Micam Ultima CMOS camera (SciMedia USA Ltd, CA, USA) at 2 kHz with a spatial resolution of 0.7x0.7 mm. Optical signals were filtered using a low-pass frequency filter at 120 Hz followed by spatial averaging (kernel 2.1 mm) and temporal averaging (kernel 1.5 ms).

Pseudo-ECG recordings across tissue preparations were recorded throughout experiments. In addition, to record activity along the moderator band and septum, unipolar electrograms were recorded from 5 locations simultaneously: 1) septum, close to the MB attachment; 2) proximal (septal) end of the MB; 3) mid-MB; 4) distal (RV) end of the MB and 5) the anterio-lateral papillary muscle. A reference electrode was positioned far from recording electrodes. All electrical recordings were acquired at 10 kHz and signals were treated by a forward-backward Butterworth filter with a 120 Hz low-pass cut-off.

Pacing protocols

Two stimuli locations were used: endocardial surface of the RV mid-wall and the MB at approximately half of its length from the septum. Measurements of activation latency at varying stimulation currents were recorded at a basic cycle length of 2 Hz from the stimulation threshold. S1S2 pacing was assessed for each pacing site, and a combined S1RV-S2MB protocol at twice the stimulation threshold. The stimulation sequence consisted of a train of 15 S1 stimuli at a basic cycle length of 2 Hz followed by S2. The S1S2 coupling interval was augmented until capture of an S2 beat to identify the effective refractory period (ERP). Coupling intervals were adjusted incrementally from the ERP six times by 5 ms, two times by 10 ms, two times by 25 ms and two increments of 50 ms.

Analyses and statistics

Optical activation time (AT) was the time of the maximal derivative of the fluorescent signal. RT (RT) was taken from a fixed level of 80% of repolarization. AT and RT of unipolar signals were the time of the minimum and maximum derivatives, respectively. VT involving the MB was defined as 3 or more re-entry cycles with uni-directional propagation along the MB with activation of the RV

free wall originating from the insertion of the MB. The incidence of VT was determined from the frequency of occurrences throughout the S1S2 pacing protocol. Statistical differences were assessed by the Wilcoxon matched-pairs signed rank test and defined by $P<0.05$.

Ionic models

Membrane electrical activity (I_m) for myocardium was based on the Mahajan-Shiferaw cell model[1] and the Aslanidi rabbit Purkinje cell model[2]. This ionic model is well suited for the study of arrhythmias and arrhythmia therapy[3]. The supplement details the changes and lists modifications for each model parameter (supplemental table 2) that were applied to adjust for modeling the effective heart size (I) of sheep within the rabbit ventricular geometry for better correspondence with experiments[4]. Here, we scale the wavelength of reentry in the sheep heart to the model, preserving the patterns of ventricular fibrillation in the smaller heart geometry[4]. Parameters for calculating the effective heart size are listed in supplement table 3. I is defined as:

$$I = Fm^{1/3} \tag{1}$$

Here, F is the maximum dominant frequency (Hz) and m is heart mass (g). For sheep, we obtain I=51.8 by applying F=10.1 Hz during ventricular fibrillation[5] and m=0.0006*M as suggested by Panfilov *et al.* 2006[4], where M=22,500g. In rabbit, I is 64% of that of sheep (I=33.2). Using the dimensionless parameter, $I/CV \times APD$, where CV is conduction velocity, we adjust APD by same ratio as I to obtain a model that preserves the frequencies achievable in the rabbit geometry as observed in sheep. Peak current conductance of Ca, ($G_{Ca,L}$) and K, (G_{Kr}) channels in the ionic models were changed to conserve APD. To define a heterogeneous model to reproduce experimental observations, APD were further decreased by 21% in MB nodes.

Cell models were subjected to 320 s of pacing at a basic cycle length of 320 ms for stabilization and the last AP was used for comparison. Initial states of ionic variables were captured 1 ms prior to stimulation of the last beat.

Supplemental table 2. Parameter modifications for each ionic model

Parameter	Myocardium	Moderator band	Purkinje
**gCa**	**x0.9**	**x0.7**	
**gKr**	**x5.0**	**x6.0**	**x5.0**
**gCaB**			**x0.9**

Modifications were applied to the Mahajan-Shiferaw model for myocardium and muscular compartment of the moderator band and to the Aslanidi rabbit cell ionic model for Purkinje fibers.

Supplemental table 3. Estimating effective heart size in tourus models

Parameter	Model #1	Model #2	Model #3
**APD_{80}**	**150**	**150**	**90**
**Travel length (mm)**	**62.83**	**31.4**	**31.4**
**S1S2 dissociation (mm)**	**15.7**	**7.9**	**7.9**
**S1S2 vulneribility window for re-entry (ms)**	**201-205**	**190-196**	**141-149**
**Frequency of re-entry (Hz)**	**4.7**	**8.7**	**7.1**
**Conduction velocity ($cm.s^{-1}$)**	**0.29**	**0.27**	**0.22**
**Equivalent travel length ratio**	**1.42**	**0.78**	**1.59**

Establishing reentry in 2-dimensional tourus geometries initiated by S1S2 stimulation was assessed for two different circumferences that mimic the short axis circumference and electrophysiological properties of sheep (model #1) and rabbit (model #2) hearts. The equivalent travel length ($\lambda/APD \times CV$) of model #1 was recovered in the smaller geometry by decreasing APD to 60%.

Ventricles and moderator band computer model

We used a realistic 3D rabbit ventricle finite element mesh with anisotropic fibers and Purkinje system that we have previously developed[6]. The model contained >3,000,000 tetrahedral elements with edge lengths on the order of 300 μm (supplemental figure 5A). A MB was manually added to the geometry (supplemental figure 5B). MB computational elements merged with the myocardial elements, thus making the MB electrically continuous with the adjoining myocardium. An additional branch of the Purkinje system was inserted that extended from the His bundle and followed the central axis of the MB to form junctions at the mid RV free wall with four intramural terminal points. PMJs were absent along the MB. To mimic experimental preparations, clipped wedge geometry was also used to compare with whole heart simulations (supplemental figures 5C and 5D).

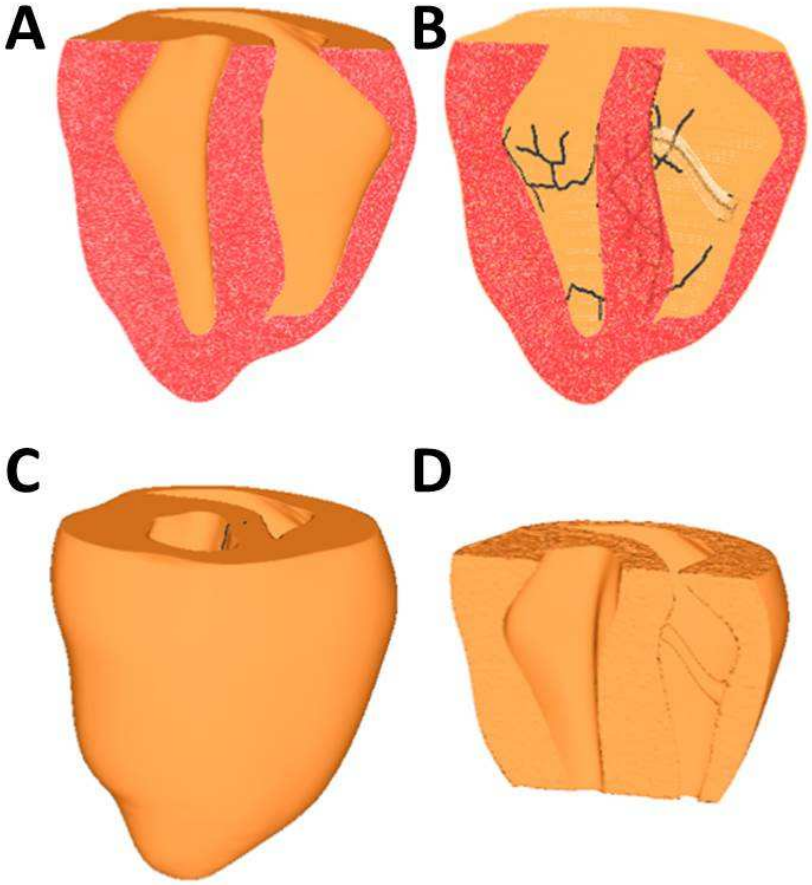

Supplemental figure 2. Three-dimensional rabbit ventricular meshes. Myocardial mesh (A) complete with Purkinje system and moderator band (B) were used for the in silico study. Simulations were implemented in whole ventricles (C) and compared to wedge preparations (D) equivalent to experiments.

We used the well-validated Purkinje-myocyte model developed by our team[6,7]. Electrical activity was solved with the CARP simulator[8] using the monodomain approach[9] with nominal extracellular conductivity values of 0.625, 0.236 and 0.236 S/m in the longitudinal (σL), transverse (σT) and transmural (σS) directions, corresponding to a conductivity anisotropy ratio of 1:0.37:0.37. The corresponding intracellular conductivities were 0.174, 0.019 and 0.019 S/m, respectively[10]. Myocardium and Purkinje fibers were pre-conditioned by states of ionic variables captured from single cell pacing during 100 beats stimuli at a basic cycle length of 320ms, determined by $I \times BCL_{expt}$ where BCL_{expt} is the basic cycle length of S1 pulses in experiments.

In silico reentry study

An S1S2 stimulus protocol was applied in the model by point stimulation of S1 pulses followed by a single S2 test pulse. S1S2 coupling intervals ranging from 100 ms were tested. Simulations were repeated while incrementing S1S2 coupling intervals by 1 ms for each iteration until a maximum of 180 ms. Re-entry was confirmed if nodes in the muscular compartment at the midpoint of the MB depolarized above a threshold of -30 mV three or more times following S2 stimuli.

Results

Structural substrate of the MB

The MB structure is composed of two excitable, yet uncoupled compartments in sheep. Purkinje fibers were separated from the myocardium in a coaxial configuration by lipid deposits and extensive extracellular collagen surrounding individual bundles, as seen in insertions (supplemental figure 3) with no apparent coupling. The Purkinje fiber/muscle ratios were highly variable, ranging from 0.04 to 0.63 (supplemental figure 4). This may be explained by a high intra-species variability of the MB thicknesses (3.3±0.9 mm, minimum=2.1 mm, maximum=5.0 mm, in sheep).

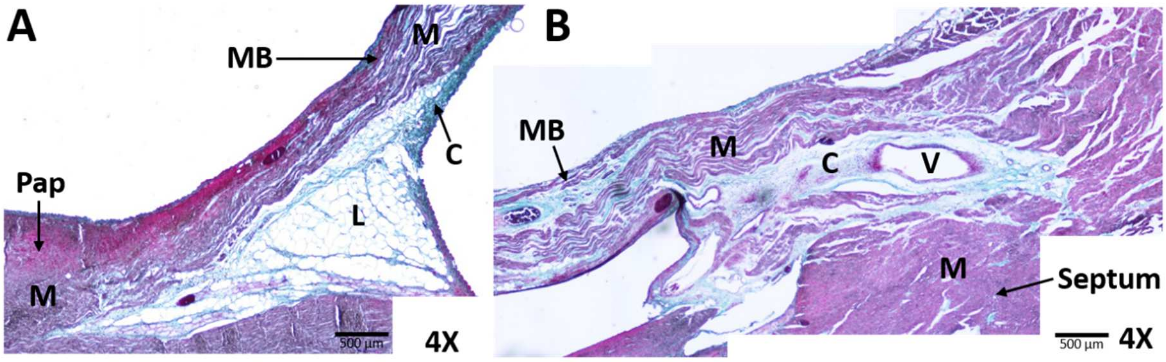

Supplemental figure 3. Structural composition of the MB insertions. Montages of MB insertions at the RV (A) and septum (B). Cellular and extracellular constituents are: collagen, C; Lipid droplets, L; myocardium, M and vasculature, V.

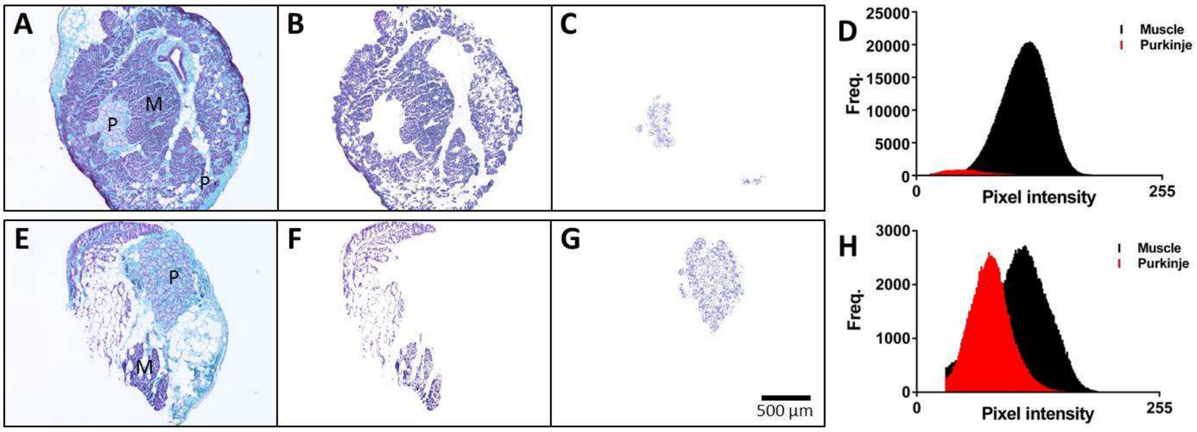

Supplemental figure 4. Composition of the moderator band. Transverse cross-sections of moderator bands were stained for myocardial and Purkinje cells, collagen and nuclei for two different moderator bands (A & B). Panels from left to right are: complete moderator band cross-sections; myocardial compartments; Purkinje compartments and frequency distributions of pixel intensity for myocardial and Purkinje compartments.

MB conduction behaviour

Conduction delays between the RV free wall and MB myocardium in sheep were dependent upon the direction of propagation across the MB insertion and the coupling interval. This was determined through S1S2 pacing of either the RV free wall or the myocardial compartment of the MB. Across all experiments, activation latency of the MB following RV stimulation at the S1 basic cycle length was 12.7±6.1 ms. This was increased to 16.0±8.3 ms at the ERP of the stimulation site. For MB stimulation, activation latency of the RV was 14.4±13.6 ms at the basic cycle length but significantly prolonged to 32.5±16.7 ms at the ERP (P<0.05). An example of short coupled propagation along the MB is shown for S1S2RV and S1S2MB pacing in supplemental figure 5.

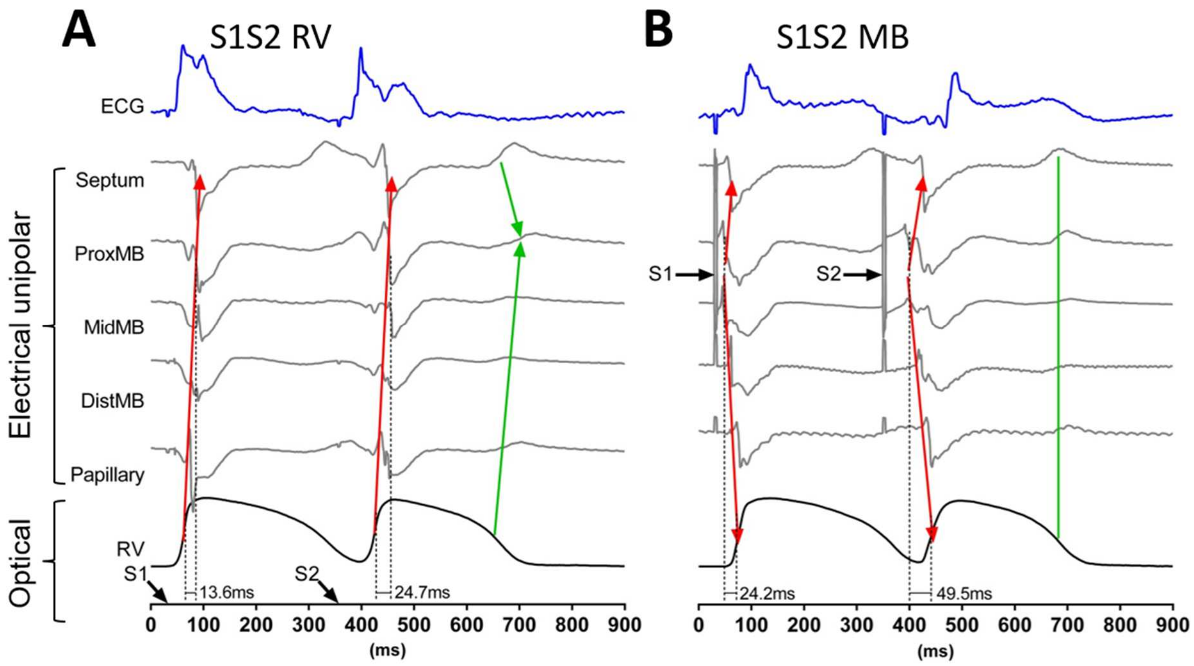

Supplemental figure 5. Short coupled S1S2 pacing intervals. The last of a train of S1 pulses and the S2 beat from the ERP were captured by unipolar recordings and simultaneous optical mapping of the RV free wall for RV (A) and MB (B) pacing (black arrows). Directions of propagation (red arrows) are shown.

Macroreentry involving the human MB

Conduction behavior and repolarization heterogeneity were examined from two human wedge preparations with intact MBs. MB thicknesses from human preparations were (4.0 and 8.5 mm in donor #1 and donor #2, respectively). Pacing at a basic cycle length of 500 ms on the MB triggered activation propagating bi-directionally towards both septal and RV free wall insertions of the MB. The activation latency of the RV when pacing MB was 10.8 ms and 17.9 ms, respectively (see

supplemental figure 6). In donor #2, a sufficiently short coupled stimulation at the same site resulted in uni-directional propagation towards the RV free wall to excite the septum from the RV free wall instead of the MB directly.

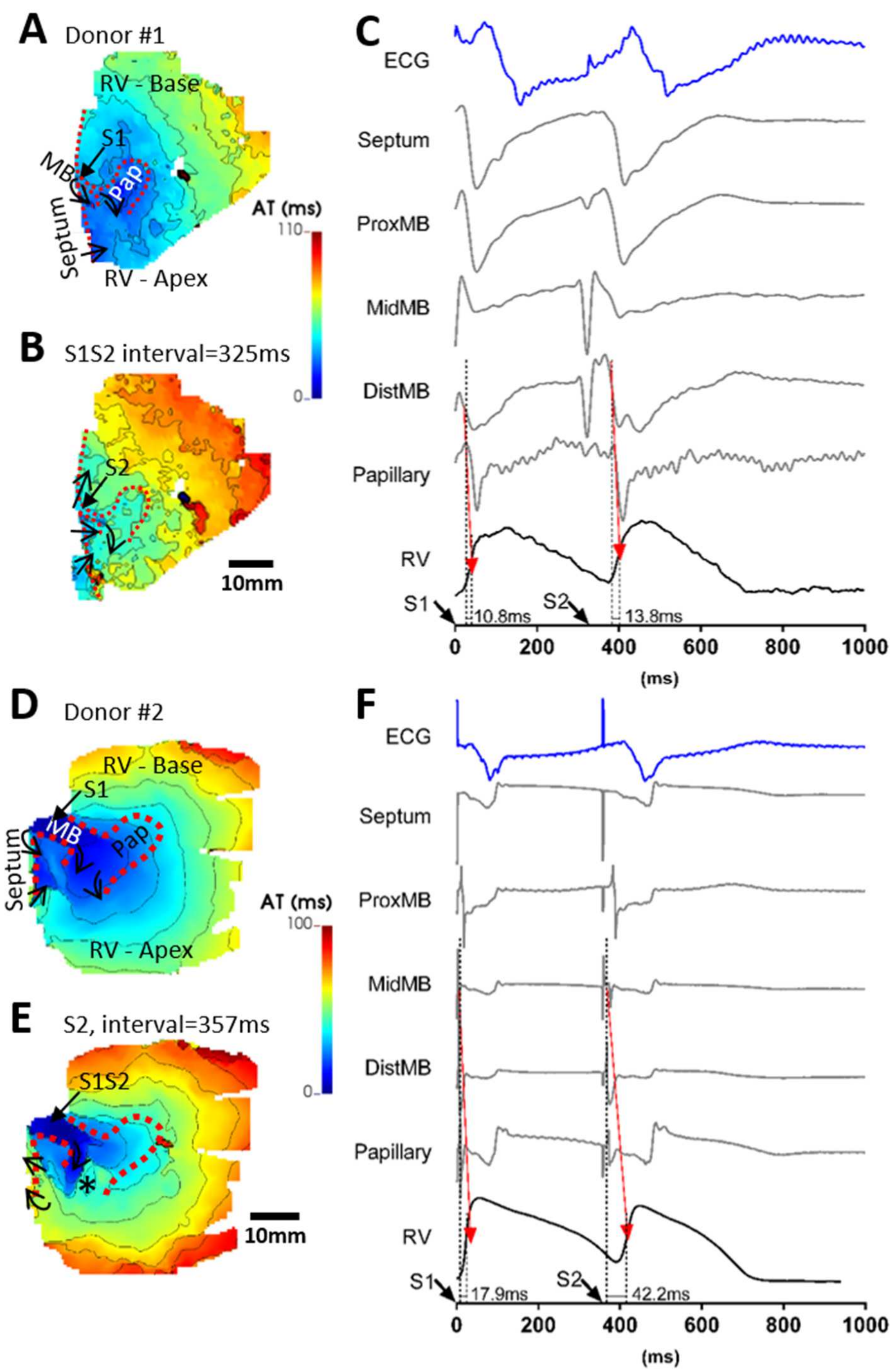

Supplemental figure 6. Short-coupled stimulation of the MB in humans. Activation patterns during pacing human donor #1 MB at A) basic cycle lengths of 500 ms and B) a short coupled beat of 325 ms. C) Unipolar electrical and optical AP traces extracted from the septum, along the MB and the RV site of early activation following the final S1 and S2 pulses. D-F) Same as for A-C for donor #2.

In silico reentry

Simulations of S1S2RV, S1S2MB and S1RV-S2MB pacing protocols are shown in supplemental videos 4-6. In each case, VT could be induced in homogeneous and heterogeneous models, but windows of vulnerability for VT were increased in the heterogeneous model (supplemental figure 7A-B). Furthermore, the direction of the reentrant circuit was reversed for S1S2RV pacing as a result of reversal of the activation and repolarization sequences of the S2 beat relative to S1S2MB and S1RV-S2MB simulations. A further model was implemented whereby the Purkinje network was omitted (supplemental figure 7C and supplemental video 7). In this case, neither S1S2RV nor S1S2MB could induce VT, indicating a predominant role of Purkinje in the induction of the VT as opposed to the muscular compartment of the MB. However, inducibility of sustained stable reentrant arrhythmias was unchanged in S1RV-S2MB simulations. To examine the impact of the wedge preparation in experiments, the whole ventricle mesh was clipped to a geometry corresponding closely to experiments and zero-boundary conditions applied to clipped surfaces. In a model with homogeneous ionic properties, windows of vulnerability for each pacing protocol were largely unchanged, particularly for S1RV-S2MB pacing (supplemental figure 7D and supplemental video 8).

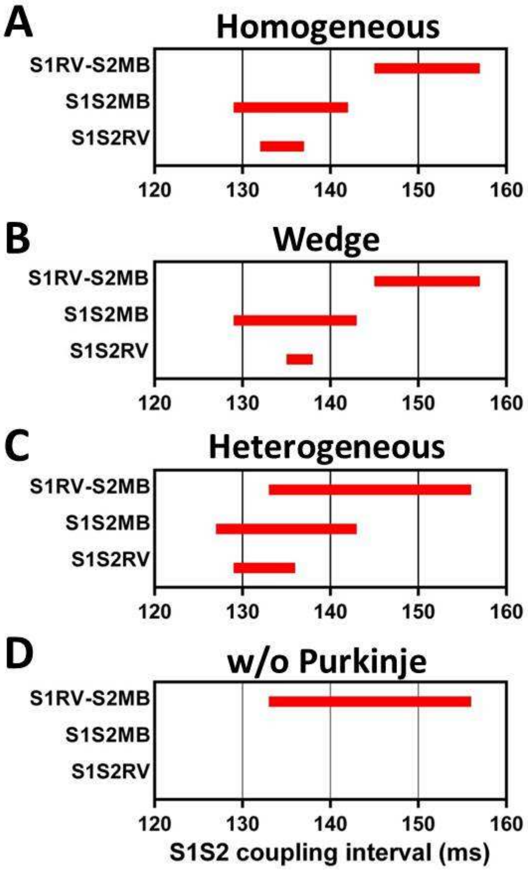

Supplemental figure 7. Windows of vulnerability for reentry in simulations. The windows of vulnerability for each stimulus configuration are shown for: A) a homogeneous whole heart model; B) wedge preparation; C) a heterogeneous model with reduced APD in the MB and D) a heterogeneous model in the absence of a Purkinje system.

Supplemental references

1. Mahajan A, Shiferaw Y, Sato D, Baher A, Olcese R, Xie LH, Yang MJ, Chen PS, Restrepo JG, Karma A, Garfinkel A, Qu Z, Weiss JN. A rabbit ventricular action potential model replicating cardiac dynamics at rapid heart rates. *Biophys J*. 2008;94:392–410. doi:10.1529/biophysj.106.98160.
2. Aslanidi O V, Stewart P, Boyett MR, Zhang H. Optimal velocity and safety of discontinuous conduction through the heterogeneous Purkinje-ventricular junction. *Biophys J*. 2009;97:20–39. doi:10.1016/j.bpj.2009.03.061.
3. Vadakkumpadan F, Rantner LJ, Tice B, Boyle P, Prassl AJ, Vigmond E, Plank G, Trayanova N. Image-based models of cardiac structure with applications in arrhythmia and defibrillation studies. *J Electrocardiol*. 2009;42:157 e1-10. doi:10.1016/j.jelectrocard.2008.12.003.
4. Panfilov A V. Is heart size a factor in ventricular fibrillation? Or how close are rabbit and human hearts? *Hear Rhythm*. 2006;3:862–864. doi:10.1016/j.hrthm.2005.12.022.
5. Moreno J, Zaitsev A V, Warren M, Berenfeld O, Kalifa J, Lucca E, Mironov S, Guha P, Jalife J. Effect of remodelling, stretch and ischaemia on ventricular fibrillation frequency and dynamics in a heart failure model. *Cardiovasc Res*. 2005;65:158–166. doi:10.1016/j.cardiores.2004.09.006.
6. Vigmond EJ, Clements C. Construction of a computer model to investigate sawtooth effects in the Purkinje system. *IEEE Trans Biomed Eng*. 2007;54:389–399. doi:10.1109/TBME.2006.888817.
7. Boyle PM, Deo M, Plank G, Vigmond EJ. Purkinje-mediated effects in the response of quiescent ventricles to defibrillation shocks. *Ann Biomed Eng*. 2010;38:456–468. doi:10.1007/s10439-009-9829-4.
8. Vigmond EJ, Hughes M, Plank G, Leon LJ. Computational tools for modeling electrical activity in cardiac tissue. *J Electrocardiol*. 2003;36 Suppl:69–74.
9. Vigmond EJ, Aguel F, Trayanova NA. Computational techniques for solving the bidomain equations in three dimensions. *IEEE Trans Biomed Eng*. 2002;49:1260–1269. doi:10.1109/TBME.2002.804597.
10. Clerc L. Directional differences of impulse spread in trabecular muscle from mammalian heart. *J Physiol*. 1976;255:335–346.

Supplemental video legends

Supplemental video 1. S1-sinus rhythm S2-MB pacing in a homogeneous whole ventricular computer model. The clipped plane was for visualization of the ventricular cavities only. S1S2 coupling interval = 133 ms.

Supplemental video 2. Macroreentrant VT induced by S1-sinus rhythm S2-MB pacing in a heterogeneous whole ventricular model. S1S2 coupling interval = 122 ms.

Supplemental video 3. Macroreentrant VT in a heterogeneous model is dependent upon the MB. S1-sinus rhythm S2-MB pacing in a heterogeneous whole ventricular model inducing VT was blocked by applying GNa=0.0 of the myocardial compartment of the MB during VT (t=300ms). S1S2 coupling interval = 122 ms.

Supplemental video 4. Macroreentrant VT induced by S1S2-RV pacing in a heterogeneous whole ventricular model. S1S2 coupling interval = 133 ms.

Supplemental video 5. Macroreentrant VT induced by S1S2-MB pacing in a heterogeneous whole ventricular model. S1S2 coupling interval = 133 ms.

Supplemental video 6. Macroreentrant VT induced by S1-RV S2-MB pacing in a heterogeneous whole ventricular model. S1S2 coupling interval = 133 ms.

Supplemental video 7. Macroreentrant VT induced by S1-RV S2-MB pacing in a heterogeneous whole ventricular model in the absence of a Purkinje network. S1S2 coupling interval = 133 ms.

Supplemental video 8. Macroreentrant VT induced by S1-RV S2-MB pacing in a heterogeneous ventricular wedge model. Clipped planes are real geometry boundaries. S1S2 coupling interval = 133 ms.
