# Supplementary figures and images for: Compartmentalized Structure of the Moderator Band Provides a Unique Substrate for Macroreentrant Ventricular Tachycardia
Source: Circ Arrhythm Electrophysiol. 2018 Jul 31;11(8):e005913. doi: 10.1161/CIRCEP.117.005913 (PMC7661046; doi:10.1161/CIRCEP.117.005913)

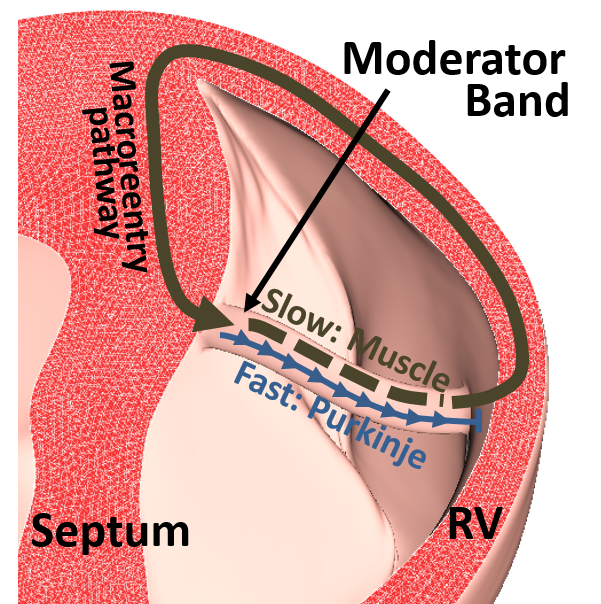

Supplement: SUPPLEMENTARY MATERIAL [file hae-11-e005913-s010.tif]
